# Health-Related Quality of Life in a Cohort of Breast Cancer Survivors over More Than 10 Years Post-Diagnosis and in Comparison to a Control Cohort

**DOI:** 10.3390/cancers13081854

**Published:** 2021-04-13

**Authors:** Tabea Maurer, Kathrin Thöne, Nadia Obi, Audrey Y. Jung, Sabine Behrens, Heiko Becher, Jenny Chang-Claude

**Affiliations:** 1 Cancer Epidemiology Group, University Cancer Center Hamburg (UCCH), University Medical Center Hamburg-Eppendorf, 20251 Hamburg, Germany; ta.maurer@uke.de (T.M.); dr.kathrin.thoene@tk.de (K.T.); 2 Institute for Medical Biometry and Epidemiology, University Medical Center Hamburg-Eppendorf, 20251 Hamburg, Germany; n.obi@uke.de (N.O.); h.becher@uke.de (H.B.); 3 Division of Cancer Epidemiology, German Cancer Research Center (DKFZ), 69120 Heidelberg, Germany; audrey.jung@dkfz-heidelberg.de (A.Y.J.); s.behrens@dkfz-heidelberg.de (S.B.)

**Keywords:** breast cancer, survivorship, longitudinal, health-related quality of life, population-based, age effects

## Abstract

**Simple Summary:**

Breast cancer survivors often experience long-term side-effects of the disease and its treatment that negatively impact their quality of life. However, to date only few long-term studies on breast cancer survivor’s quality of life exist and it is unclear whether or not breast cancer survivors experience a worse quality of life than women without breast cancer. We therefore investigated breast cancer survivor’s quality of life before diagnosis, during active treatment as well as 5 and 10 years after diagnosis and compared it to the quality of life in women without breast cancer. We found that breast cancer survivor’s quality of life over all ages improved in the first 5 years and then started to deteriorate. After 10 years it was comparable to women without breast cancer. Yet, we showed that survivors of different ages experience differences in health related quality of life over time. Most importantly, we showed that 10 years after diagnosis younger patients reported a worse quality of life than women of the same age that never had breast cancer. These findings are important when trying to optimize long-term care of breast cancer survivors.

**Abstract:**

**Background:** Breast cancer (BC) survivors often suffer from late and long-term residual symptoms of the disease and its treatment. To date, long-term health-related quality of life (HRQoL) in breast cancer survivors has been seldom investigated and rarely compared to unaffected women (controls). **Aim:** This study aimed to investigate HRQoL over time using patient-reported status before diagnosis, during treatment, 1 year post-surgery, approx. 5 years and ≥10 years post-diagnosis. We also compared survivors’ HRQoL with controls’ still alive 10 years after recruitment. **Methods:** Data from the German population-based Mamma Carcinoma Risk Factor Investigation (MARIE) cohort of 1123 BC patients aged 50–74 years at diagnosis (2002–2005) and of 3453 matched controls were used for analysis. HRQoL was assessed with the European Organization for Research and Treatment of Cancer (EORTC QLQ-C30) questionnaire. All analyses were conducted for all ages as well as stratified according to three age groups (≤58 years, 59–64 years, ≥64 years). Differences in survivors’ general HRQoL before, during, and after therapy were investigated using a t-test/Wilcoxon signed-rank test. Changes in the HRQoL of survivors stratified by age from FU1 to FU2 were assessed via repeated analysis of variance. The HRQoL of survivors compared to the controls at FU2 was analyzed using an analysis of variance. **Results:** Over all ages, the general HRQoL in patients improved in the first 5 years post-diagnosis. In the subsequent years, HRQoL slightly deteriorated but was comparable to that of the controls. Younger survivors mostly improved their HRQoL from the 5 to 10-year follow-up but remained negatively affected for most functioning and symptom scales compared to controls. In older survivors, HRQoL hardly changed over time and detriments were less pronounced compared to controls, except for insomnia. **Conclusions:** Restrictions of HRQoL persist for more than 10 years and are most prominent among younger survivors. Researchers and clinicians should be aware of such potential deteriorations and age-dependent differences in order to optimize/adapt long-term cancer survivor care.

## 1. Introduction

With almost 70,000 incident cases annually, breast cancer represents one of the most common malignancies among women in Germany [1]. The number of women diagnosed with breast cancer is also expected to increase due to demographic aging [2]. At the same time, due to improvements in the modes of detection and targeted treatment, breast cancer mortality is likely to continue decreasing over the coming decades [3]. Consequently, the number of long-term cancer survivors will continue to increase. However, many breast cancer survivors suffer from negative sequela, such as physical and mental health issues, after a cancer diagnosis and its treatment—even decades thereafter. Long-term health effects after a cancer diagnosis are multifactorial and comprise chronic diseases such as osteoporosis, hypertension, heart failure, diabetes, or dementia [4] as well as treatment-related persisting effects such as fatigue, depression, sleep disorders, or cognitive dysfunction [5,6]. Such comorbidities and late complications have an impact on health-related quality of life (HRQoL), which itself is a multi-dimensional concept related to physical, mental, emotional, and social functioning [7]. Consequently, research to determine the effects of chronic illnesses and their treatment, as well as the associated short and long-term disabilities, on HRQoL in cancer survivors has become of great interest.

Several studies reported that the general HRQoL of cancer patients—e.g., at diagnosis [8], 1 year after diagnosis [9], 3 years after diagnosis [10], 5 years after diagnosis [11,12] and 10 years after diagnosis [13,14], is comparable with that of the general population. However, studies on long-term survivorship (≥5 years post-diagnosis) are rare and their findings for HRQoL subdomains (functioning and symptoms) as well as the potential differences according to age at diagnosis are inconsistent [13,14,15,16,17]. This is the first study to investigate the course of HRQoL in patients with more than 10 years of follow-up, stratified by age, and with the aim to identify QoL restrictions that may differentially impact age groups over time. In addition, QoL in patients is compared to age-matched cancer-free controls similarly followed up for 10 years to investigate whether long-term QoL issues are related to the diagnosis and cancer treatment rather than to ageing.

## 2. Methods

### 2.1. Data Source and Study Population

We used data from patients and control women who initially participated in the population-based case-control study Mamma Carcinoma Risk factor Investigation (MARIE) [18], which has been transformed into patient and control cohort studies through continued follow-ups of the participants approximately every 5 years. 

Initially, patients (cases) aged 50–74 years with a histologically confirmed diagnosis of primary invasive (stage I to IV) or in situ breast cancer between 1 January 2001, and 30 September 2005, were recruited from two study regions in Germany, Hamburg, and Rhine-Neckar-Karlsruhe. Women without breast cancer diagnosis (controls) were drawn from the population registries and the frequency was matched by birth year and the study region to the cases (ratio two-to-one). At recruitment, 3813 cases and 7341 controls completed a standardized face-to-face interview. Information on pre-diagnostic lifestyle factors, socioeconomic status, medical history, as well as specific medications, regimen, and duration of use was collected. The histological characteristics of the primary breast cancer were extracted from pathology reports. Treatment and clinical course were abstracted from medical records to verify clinical events either self-reported in the follow-up interview or reported by treating physicians. 

Cases were re-contacted for a first follow-up (FU1) in 2009 and a second follow-up (FU2) in 2014/2015, whereas that of the controls was in 2011/2012 and 2016, respectively. The end of follow-up time was death, emigration, or last contact up to the date of censoring (30 June 2015, for cases and 31 December 2016, for controls). For the cases, information on current HRQoL was collected at FU1 and FU2 using the questionnaire provided by the European Organization for Research and Treatment of Cancer (EORTC QLQ-C30). At FU1, cases were also asked retrospectively for their HRQoL pre-diagnosis, during treatment phases, and 1 year post-surgery since HRQoL data were not collected at the baseline. For controls, information on HRQoL was collected only at FU2. 

All study participants gave written informed consent. The ethics committee of the University of Heidelberg, the Hamburg Medical Council, and the Medical Board of the State of Rhineland-Palatinate gave approval. The study was conducted in accordance with the Declaration of Helsinki. 

Overall, 510 (13.4%) and 392 (11.9%) study cases deceased by FU1 and FU2, respectively, 11 cases were lost to follow-up, and 5 emigrated. Information on HRQoL was available for 2326 (61%) at FU1 and 1725 (45%) at FU2. Patients were excluded if they had malignant tumors other than BC prior to baseline *(N =* 85), presented with tumors stage IIIb or higher (*N =* 181), developed recurrences (*N* = 59), metastases (*N =* 29), or secondary tumors (*N* = 89). All cases included in the study population are defined as “full responders” if they gave information on HRQoL at FU1 and FU2. Controls were excluded if HRQoL data were missing at FU2 (*N* = 3692) or if controls were diagnosed with breast cancer during follow-up (*N* = 95 until FU1, *N* = 101 between FU1 and FU2). Thus, data from 1123 cases and 3453 controls were available for analysis. Cases were defined as “partial responders” if they provided information on HRQoL at only one of the two time-points (*N* = 481): either FU1 (*N* = 371) or FU2 (*N* = 110). 

### 2.2. Health-Related Quality of Life Measurement 

The EORTC QLQ-C30 questionnaire was specifically designed for cancer patients. Reference values are available in a healthy control population for multiple countries, including 11 European Union countries (i.a. Germany) [19]. The validated manual consists of 30 items and is composed of a 2-item general health/HRQoL scale as well as 5 multi-item function scales to assess the physical, role, social, emotional, and cognitive functions; three multi-item symptom scales to assess fatigue, pain, and nausea/vomiting; and six single-items that assess symptoms such as dyspnea, insomnia, appetite loss, constipation, diarrhea, and financial difficulties. In accordance with the guidelines provided by the EORTC, all scores of the QLQ-C30 were transformed linearly so that all the scales ranged from 0 to 100 [7]. Mean imputation of missing data was performed if less than half of the questions used to calculate the respective score were missing. High scores in the function scales represent a better level of functioning and on the general health/HRQoL scale a better overall HRQoL, while in the symptoms scales/items higher scores represent a higher level of symptoms or problems. The clinical relevance of the differences between different time points or between cases and controls were interpreted qualitatively according to Cocks and King [20]. Based on a meta-analysis of 118 papers and one thousand two hundred and thirty two mean changes in QOL over time, guidelines were produced for trivial, small, and medium-size classes for each subscale and the improving and declining scores separately [20].

### 2.3. Statistical Analysis

All analyses were conducted for all ages as well as stratified according to three different age groups (≤58 years, 59–64 years, ≥64 years). Differences in the survivors’ general HRQoL before, during, and after therapy were investigated using the t-test/Wilcoxon signed-rank test. Changes in the HRQoL in survivors stratified by age from FU1 to FU2 were investigated via repeated analysis of variance. The HRQoL of survivors compared to the controls at FU2 was analyzed using analysis of variance. All tests were two-sided and a *p*-value of less than 0.05 was considered statistically significant. Analyses were performed using the SAS statistical software, Version 9.4 (SAS Institute Inc., Cary, NC, USA). 

## 3. Results

For descriptive purposes, cases and controls, as well as full and partial responders among cases, were compared using age at diagnosis or recruitment, parenthood status, education status, family status, smoking status, alcohol intake, BMI, physical activity, osteoporosis, diabetes, cardiovascular diseases, and rheumatic diseases (Table 1).

### 3.1. Longitudinal Analysis of HRQoL in Breast Cancer Survivors and Comparison with Controls

#### 3.1.1. General HRQoL 

**Figure 1:**
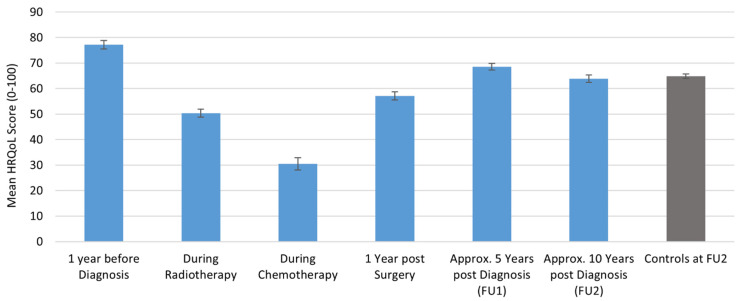
Longitudinal course of general HRQoL in breast cancer survivors over all age groups and compared to controls at approx. 10 years after baseline assessment.

For cancer survivors, the general HRQoL for all ages was highest one year before diagnosis (baseline) and lowest during chemotherapy. HRQoL increased one year after surgery, but was still significantly lower than before diagnosis (mean_diff_ = −20.08, 95%CI = −21.97–−18.20, *p* = < 0.0001). At FU1, approx. 5 years after diagnosis, HRQoL further increased, but remained significantly lower than at the baseline (mean_diff_ = −8.57, 95%CI = −10.201–6.921, *p* = < 0.0001). At FU2, approx. 10 years post-diagnosis breast cancer survivors’ HRQoL slightly decreased, but levels were comparable to healthy controls at FU2 (mean_diff_ = −0.90, 95%CI = −2.63–0.83, *p* = 0.31) (Figure 1).

**Figure 2:**
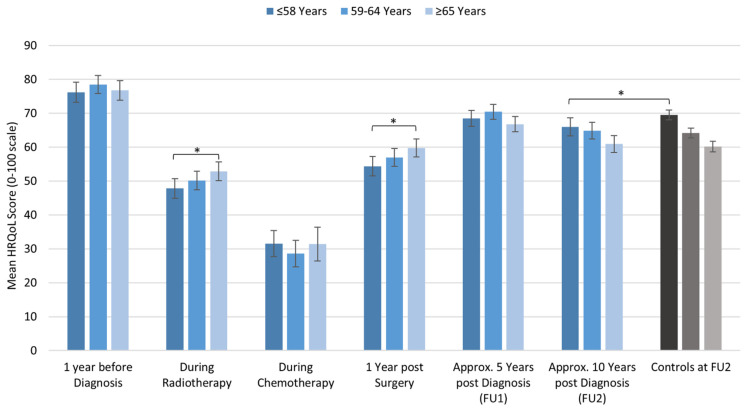
Longitudinal course of HRQoL in breast cancer survivors and compared to controls at approx. 10 years after diagnosis stratified by age. Significant differences are indicated by an asterisk.

There were differences in the general HRQoL between age groups. During radiation therapy and one year after surgery, younger survivors reported significantly lower HRQoL than patients in the oldest age group. Approx. 10 years after diagnosis, older patients experienced the most prominent deterioration. Compared to healthy controls at that point in time, only younger patients had significantly lower HRQoL scores (Figure 2 and Table 2).

#### 3.1.2. Cases’ Long-Term Course of Functioning Scales Stratified by Age and Compared to Healthy Controls

**Figure 3:**
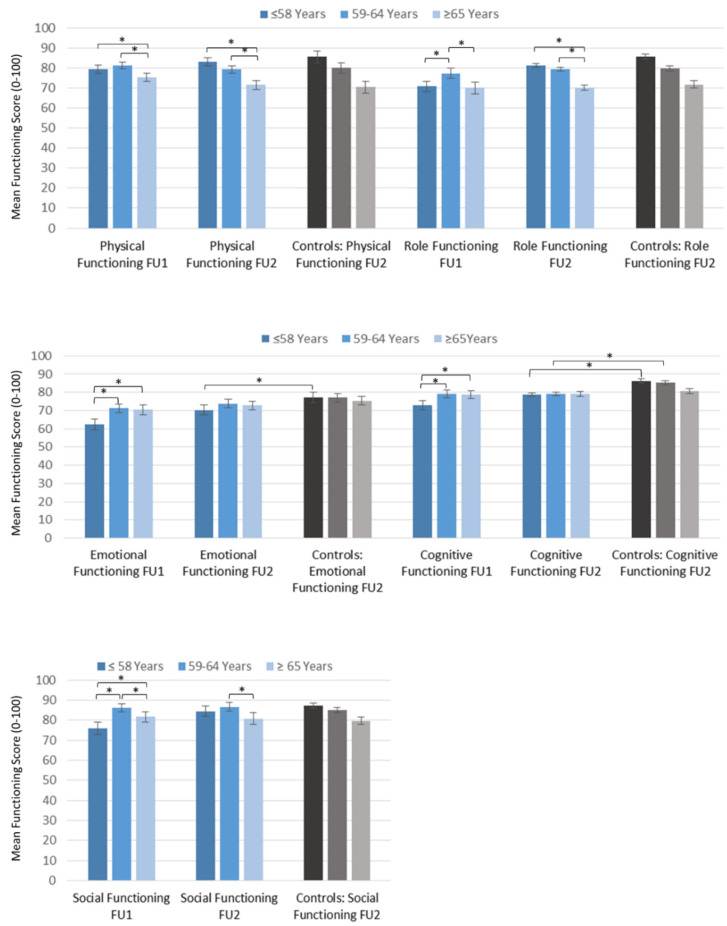
Functioning in breast cancer survivors at FU1 and FU2 stratified by age and compared to controls. Significant differences are indicated by an asterisk.

At FU1 the younger and middle-aged group scored higher on the physical functioning scale than older patients. At FU2 younger patients further improved their physical functioning while older patients’ physical functioning further decreased. Compared to the age-matched population controls there were no differences in any of the age groups (Table 3 and Figure 3).

On the emotional and cognitive scale assessed at FU1, younger survivors scored lower than the middle and older age group but increased their scores at FU2, whereas older survivors’ scores did not change. Compared to healthy controls at FU2, younger survivors scored worse on both scales. Survivors of the middle age group showed comparable emotional functioning but had cognitive deficits compared to controls. No differences were found on either scale between the older survivors and older controls (Table 3 and Figure 3).

For social and role functioning at FU1, younger and older survivors reported lower scores than middle-aged patients. Younger patients increased both scores at FU2 while older and middle-aged patients showed no differences compared to FU1. There were no differences between survivors and controls at FU2 (Table 3 and Figure 3).

#### 3.1.3. Survivors’ Long-Term Course of Symptom Scales Stratified by Age and Compared to Healthy Controls

**Figure 4:**
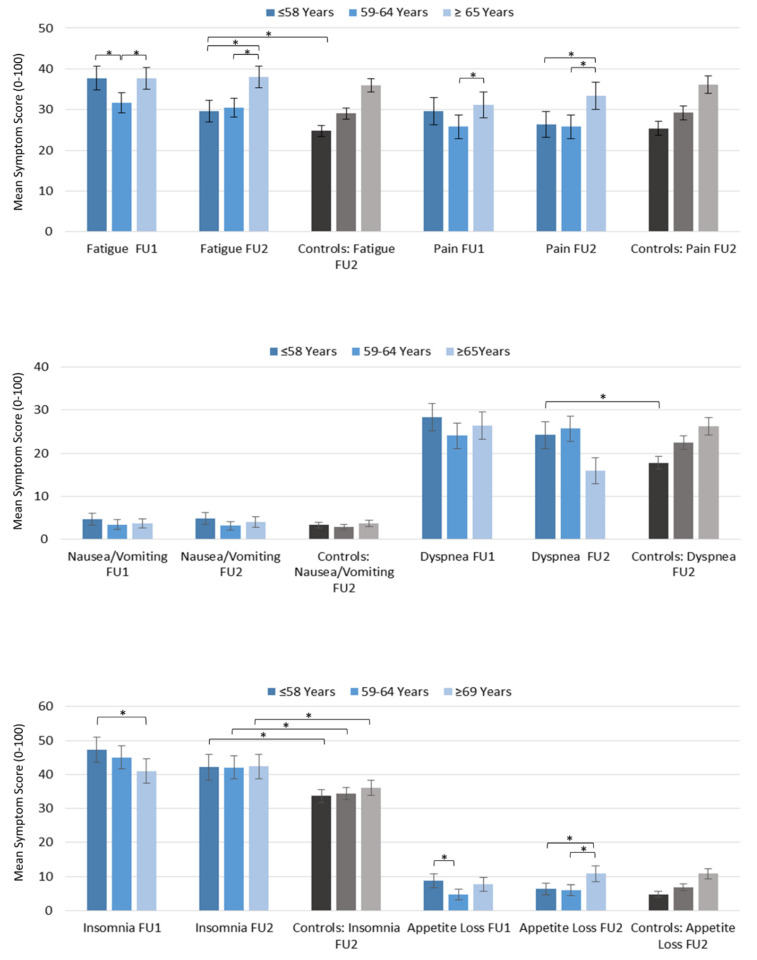

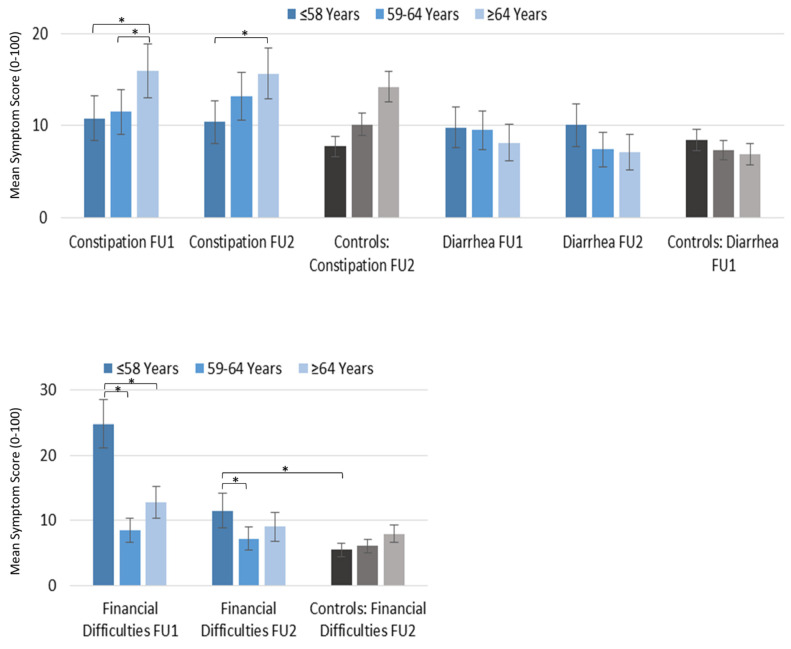
Symptoms in breast cancer survivors at FU1 and FU2 stratified by age and compared to controls. Significant differences are indicated by an asterisk.

At FU1, younger and older survivors were more fatigued than the middle age group. At FU2, fatigue scores in younger patients significantly decreased. Middle and older aged patients at FU2 showed no differences to FU1. Compared to the healthy controls, only younger patients were more fatigued at FU2 (Table 4 and Figure 4). 

At FU1, patients of all age groups reported similar pain levels. At FU2, younger patients’ pain levels decreased while there were no significant changes in the middle and older patients. There were no differences compared to the controls at FU2 (Table 4 and Figure 4).

For nausea and vomiting, diarrhea, dyspnea, and insomnia, there were no differences between age groups at FU1 or FU2. For younger patients, dyspnea and insomnia decreased from FU1 to FU2. No changes were found in older aged patients. Compared to the healthy controls, only younger patients reported stronger symptoms of dyspnea while insomnia patients in all age groups had a significantly higher dyspnea symptom burden. For nausea and vomiting, and diarrhea, survivors of all ages were comparable to controls (Table 4 and Figure 4). 

At FU1, younger patients had significantly less appetite than both older age groups. At FU2, in younger survivors, this symptom decreased while older survivors reported significantly less appetite compared to FU1. All age groups were comparable to healthy controls at FU2 (Table 4 and Figure 4).

Constipation at FU1 was most pronounced in older patients and was not significantly reduced at FU2. Compared to healthy controls there were no differences for any age group at FU2 (Table 4 and Figure 4).

At FU1, financial difficulties were significantly stronger in younger than older survivors. From FU1 to FU2 younger patients’ financial difficulties were reduced, but remained significantly worse compared to controls. Middle and older-aged patients’ financial difficulties hardly changed over time and were comparable to the controls (Table 4 and Figure 4).

## 4. Discussion 

We found improvements in the general HRQoL in long-term breast cancer survivors of all ages in the first 5 years after the completion of treatment. Even though the HRQoL never returned to pre-diagnosis levels and further deteriorated slightly over the subsequent ≥5 years, cancer survivors were comparable to controls at about 10 years post recruitment. However, differences in the general HRQoL during and after treatment were found when stratifying by age. Younger survivors experienced greater detriments in general HRQoL during radiation as well as one-year post-surgery and remained burdened compared to the controls at 10 years after the diagnosis. Interestingly, younger survivors improved on most functioning scales and experienced a reduction in most symptoms over time, whereas older survivors showed no change or a worsening in symptoms and functioning. Yet, compared to controls of comparable age that were still alive after more than 10 years, younger patients reported clinically meaningful poorer cognitive and emotional functioning as well as clinically meaningful stronger symptoms of insomnia, dyspnea, fatigue, and financial difficulties whereas older survivors were comparable to the controls on all scales except for a higher burden of insomnia. 

### 4.1. Longitudinal Development of HRQoL in Long-Term Breast Cancer Survivors

The few longitudinal studies of QoL in long-term breast cancer survivors (≥5 years follow-up) found improvements or stability in general HRQoL and most QoL domains over time [12,15,17,21,22]. Only two studies reported an increase in physical symptoms [21] and bodily pain [17] as well as a decline in physical functioning, role function, and general health; however, the effects were modest and could be explained by the general aging process [17]. These results are mostly in line with our study; however, the comparability is limited as patients were not stratified by age and follow-up times differed. The only other longitudinal study was taken from 160 patients that were aged 18 years or older and analyzed assessments at 1, 3, 5, and 10 years post-diagnosis stratified by age. The study found a steady decline in QoL and reported aggravating detriments in various QoL dimensions (e.g., physical, role, cognitive and social functioning; pain, fatigue, appetite loss, constipation, diarrhea, and financial difficulties) from years 5 to 10 over all ages [13]. Only emotional and social functioning increased, from 1 to 3/5 years, and was followed by a decrease until 10 years post-diagnosis. Contrary to our findings, they did not observe differences for subgroups according to age. The differing results could be due to differences in study design between the two studies. Our study included older patients, thus yielding different age categories and a much larger patient sample and therefore larger subsamples per age category.

### 4.2. Long-Term HRQoL in Breast Cancer Survivors Compared to Healthy Controls

Most studies suggest a comparable general HRQoL between long-term breast cancer survivors and controls [12,13,14,15,23]. However, in breast cancer survivors clinically significant detriments have been found for several QoL dimensions, most commonly for cognitive [12,13,14,15,16] and emotional functioning [13,14] as well as symptoms of fatigue [12,13,14,16], insomnia [12,13,14], and financial difficulties [13,14,15]. Noteworthy and not without controversy are the findings regarding a differing impact of the cancer diagnosis and its treatment on HRQoL depending on the patient’s age at diagnosis. In line with our findings, some previous studies have shown that restrictions in QoL predominantly affect younger survivors [21,24,25,26,27]. However, few studies have investigated the long-term HRQoL after more than 5 years post-diagnosis compared to controls. A German longitudinal study with up to 10-year follow-ups reported detriments for cognitive and social functioning and stronger symptoms of fatigue and dyspnea compared to the controls, especially for younger survivors compared to controls of the same age [13]. Another study, comparing younger and older breast cancer survivors three to eight years post-diagnosis to age-matched controls, found that younger patients reported more depressive symptoms and fatigue, poorer self-reported attention function, and poorer sexual function [16]. Yet, in a recent cross-sectional study of long (5–9 years post-diagnosis) and very long-term (≥10 years post-diagnosis) breast cancer survivors, differences between the patients and controls were found only in younger patients <10 years post-diagnosis [14]. For very long-term survivors, restrictions other than financial difficulties were found only in the middle (60–69 years) or older-aged (70–79 and 80–89 years) patients, whereas younger patients were comparable to controls. By using controls matched by birth year and followed up over the same time period, our findings strongly reject the differences between survivors and controls concerning the birth cohort and secular trends when compared to other studies. Albeit detriments in the general HRQoL between younger breast cancer survivors compared to their age-matched controls in our study were mostly small [20], these differences referred to HRQoL that was assessed at more than 10 years post-diagnosis. Disadvantages for younger patients could have been more pronounced at earlier stages of survivorship [14]. Long-lasting disadvantages—such as cognitive and emotional dysfunction, fatigue, as well as insomnia—may permanently inhibit a patient’s ability to return to a pre-cancer lifestyle or continue a pre-cancer career, therefore leading to further barriers such as the financial difficulties observed in younger survivors [28]. Our data showed that the middle-aged and older survivors compared to controls also still suffer from increased insomnia even after more than 10 years post-diagnosis, underpinning insomnia as a persistent problem in cancer survivors [29].

### 4.3. Strength and Limitations

To the best of the authors’ knowledge, this is the first study to investigate HRQoL in a longitudinal assessment over more than 10 years by stratifying by age and comparing to controls matched by birth year. Thus, the results of our study add important information to the existing knowledge of the HRQoL of long-term breast cancer survivors. A limitation of our study is that the pre-diagnosis HRQoL and HRQoL during treatment were assessed retrospectively. We cannot exclude the possibility of a response shift bias, which is defined as an adaptation process after a life-threatening serious disease involving changing internal standards, values, and the conceptualization of quality of life [30]. A response shift might lead to a higher rated HRQoL among survivors and differences found in our study between cases and controls might even underestimate the persisting problems. There might be a selection bias by only including the survivors participating in both follow-ups. Therefore, we investigated the HRQoL among survivors who gave information on their HRQoL either approx. 5 years post-diagnosis or more than 10 years post-diagnosis and compared their HRQoL to that of full responders as well as the controls. At both FU1 and FU2, only older partial responders scored significantly lower than full responders of the same age and experienced poorer HRQoL compared to controls at FU2 (data not shown). These differences in HRQoL between full and partial responders are to be expected from a healthy participant effect and were likely to have also been present in the controls if we had collected their HRQoL data at several time points. In addition, these analyses did not account for other characteristics—such as comorbidities, lifestyle, study region—which could explain, in part, the observed differences in the HRQoL between groups.

## 5. Conclusions

While the general HRQoL in long-term breast cancer survivors is stable or even improves over time and is comparable to that of population controls, several QoL domains are persistently affected by breast cancer and its treatment. Our findings confirm long-lasting detriments to survivors’ emotional and cognitive functioning as well as persistent symptoms of insomnia, fatigue, and financial difficulties. Of importance, they provide further evidence that the persistent restrictions in the HRQoL among older survivors are partly associated with the normal aging process, whereas restrictions in younger patients are more likely to be partly attributable to the breast cancer disease and/or its treatment. Consequently, researchers and clinicians should be aware of such potential deteriorations and age-dependent differences in order to optimize/adapt different health care needs and psychological support for long-term breast cancer survivors beyond routine care.

## Figures and Tables

**Table 1:** Descriptive characteristics of cases, controls and partial responders *.

		Controls		Cases (Full-Responders *)		Cases (Partial Responders *)	
		*n*	%	*n*	*%*	*n*	*%*
Age at Diagnosis/Recruitment	≤58	1165	(33.7)	350	(31.2)	114	(23.7)
	59–64	1288	(37.3)	395	(35.2)	171	(35.6)
	≥65	1000	(29.0)	378	(33.7)	196	(40.7)
Stage (S)	1			619	(55.1)	266	(55.3)
	2a/2b			447	(39.8)	191	(39.7)
	3a			57	(5.1)	24	(5.0)
Grading (G)	Low			253	(22.5)	138	(28.7)
	Moderate			621	(55.3)	252	(52.4)
	High			245	(21.8)	90	(18.7)
Nodal Status (N)	0			314	(67.4)	378	(78.6)
	1–3			104	(22.3)	86	(17.9)
	4–9			48	(10.3)	17	(3.5)
Tumor Size (T)	<2 cm			86	(18.5)	305	(63.4)
	2–4 cm			102	(21.9)	165	(34.3)
	≥5 cm			172	(36.9)	11	(2.3)
Education Status	Low	1749	(50.7)	625	(55.7)	280	(58.2)
	Medium	1075	(31.1)	308	(27.4)	136	(28.3)
	High	629	(18.2)	190	(16.9)	65	(13.5)
BMI at Diagnosis/Recruitment	<22.5	815	(23.6)	260	(23.2)	109	(22.7)
	22.5–<25	894	(25.9)	326	(29.0)	121	(25.2)
	25–<30	1190	(34.5)	393	(35.0)	178	(37.0)
	≥30	549	(15.9)	144	(12.8)	73	(15.2)
Smoking status	Non-smoker	1803	(52.2)	607	(54.1)	247	(51.4)
	Ex-Smoker	1098	(31.8)	340	(30.3)	124	(25.8)
	Current-smoker	552	(16.0)	176	(15.7)	110	(22.9)
Alcohol Intake per Day (gram)	0	581	(16.8)	207	(18.4)	112	(23.3)
	>0–<19	2311	(66.9)	739	(65.8)	298	(62.0)
	≥19	560	(16.2)	176	(15.7)	71	(14.8)
Physical Activity **	1	620	(18.0)	211	(18.8)	100	(20.8)
	2	654	(18.9)	222	(19.8)	111	(23.1)
	3	706	(20.4)	221	(19.7)	86	(17.9)
	4	686	(19.9)	235	(20.9)	88	(18.3)
	5	766	(22.2)	227	(20.2)	87	(18.1)
Family Status	Married	2420	(70.1)	787	(70.1)	307	(63.8)
	Single	185	(5.4)	65	(5.8)	24	(5.0)
	Separated	52	(1.5)	13	(1.2)	7	(1.5)
	Divorced	383	(11.1)	125	(11.1)	64	(13.3)
	Widowed	413	(12.0)	132	(11.8)	79	(16.4)
Parous	Yes	2932	(84.9)	932	(83.0)	409	(85.0)
	No	521	(15.1)	191	(17.0)	72	(15.0)
Osteoporosis	Yes	340	(9.8)	109	(9.7)	67	(13.9)
	No	3012	(87.2)	993	(88.4)	396	(82.3)
Diabetes	Yes	160	(4.6)	53	(4.7)	47	(9.8)
	No	3288	(95.2)	1068	(95.1)	434	(90.2)
CVD	Yes	1458	(42.2)	496	(44.2)	252	(52.4)
	No	1995	(57.8)	627	(55.8)	229	(47.6)
Rheumatic Diseases	Yes	1675	(48.5	551	(49.1)	236	(49.1)
	No	1752	(50.7)	564	(50.2)	252	(52.4)

* Partial responders returned EORTC questionnaires at only one follow-up (FU1 or FU2), full responders returned EORTC questionnaires at both follow-ups. ** Physical activity in quintiles of MET hours/week.

**Table 2:** HRQoL in breast cancer survivors during and after active treatment compared by age groups (1: ≤58 years, 2: 59-64 years, 3: ≥64 years) and compared to healthy controls of the same age group 10 years after diagnosis.

	During Radiation		During Chemotherapy		1 Year Post OP		5 Years Post OP		10 Years Post Diagnosis	
Age Groups	Mean ∆ (95%CI)	*p*	Mean ∆ (95%CI)	*p*	Mean ∆ (95%CI)	*p*	Mean ∆ (95%CI)	*p*	Mean ∆ (95%CI)	*p*
1 vs. 2	–2.34 (−7.15, 2.47)	0.49	2.94 (−3.71, 9.59)	0.55	−2.59 (−7.20, 2.03)	0.39	−1.91 (−576, 1.93)	0.83	1.06 (−3.22, 5.35)	0.83
1 vs. 3	−5.05 (−9.87, −0.22)	0.04	0.14 (−7.23, 7.51)	1.00	−5.42 (−10.07, −0.78)	0.02	1.75 (−2.13, 5.63)	0.18	5.04 (0.69, 9.38)	0.02
2 vs. 3	−2.70 (−7.36, 1.95)	0.36	−2.80 (−10.36, 4.75)	0.66	−2.84 (−7.36, 1.69)	0.30	3.66 (−0.11, 5.76)	0.69	3.98 (−0.24, 8.19)	0.07
1 vs. controls									−3.52 (−6.51, 0.52)	0.02
2 vs. controls									−0.76 (−3.70, 2.18)	0.60
3 vs. controls									−0.77 (−3.73, 2.20)	0.62

**Table 3:** Functioning of breast cancer survivors at FU1 and FU2 stratified by age and compared to healthy controls at FU2.

	Age	FU1	FU2	Controls FU2	Cases FU1 vs. Cases FU2			Cases FU2 vs. Controls FU2		
	Years	Mean	Mean	Mean	Mean ∆ (95%CI)	*p*	CR *	Mean ∆ (95%CI)	*p*	CR
Physical	≤58	79.28	83.10	85.54	3.82 (2.18, 5.47)	<0.0001	t	−2.44 (−4.53, −0.36)	0.33	
	59–64	81.49	79.27	80.12	−2.22 (−3.69, −0.74)	0.004		−0.80 (−3.00, 1.41)	0.98	
	≥64	75.19	71.59	70.65	−3.59 (−5.39, −1.79)	<0.0001	t	0.5 (−3.52, 1.83)	0.98	
Role	≤58	70.70	81.27	85.73	10.57 (7.66, 13.48)	<0.0001	s	−4.47 (−7.31, −1.63)	0.071	
	59–64	77.31	79.42	79.90	2.10 (−0.47, 4.67)	0.07		−0.51 (−3.52, 2.49)	0.1	
	≥64	70.27	69.98	72.24	−0.29 (−3.05, 2.47)	0.69		−2.19 (−5.78, 1.40)	0.76	
Emotional	≤58	62.33	70.35	77.30	8.00 (5.54, 10.48)	<0.0001	s	−6.95 (−9.72, −4.17)	<0.0001	s
	59–64	71.29	73.78	77.15	2.49 (0.28, 4.71)	0.03		−3.37 (−5.95, −0.78)	0.12	
	≥64	70.44	72.76	75.77	2.33 (0.12, 4.54)	0.03		−3.00 (−5.83, −0.17)	0.27	
Cognitive	≤58	72.96	78.67	86.33	5.71 (3.30, 8.13)	<0.0001	s	−7.66 (−10.10, −5.22)	<0.0001	m
	59–64	79.25	79.12	85.39	0.13 (−2.08, 1.82)	0.86		−6.27 (−8.56, −3.97)	<0.0001	s
	≥64	78.68	79.41	81.09	0.74 (−1.43, 2.90)	0.33		−1.86 (−4.44, 0.71)	0.67	
Social	≤58	75.84	84.54	87.51	8.70 (6.06, 11.33)	<0.0001	m	−2.99 (−5.83, −0.13)	0.40	
	59–64	86.31	86.56	85.19	0.26 (−1.97, 2.48)	0.82		1.40 (1.38, −1.40)	0.94	
	≥64	81.81	80.73	80.05	−1.08 (−3.84, −1.69)	0.74		0.78 (−2.66, 4.22)	0.1	

* Clinical relevance (CR): trivial (t), small (s), medium (m).

**Table 4:** Symptoms of breast cancer survivors at FU1 and FU2 stratified by age and compared to healthy controls at FU2.

	Age	FU1	FU2	Controls FU2	Cases FU1 vs. Cases FU2			Cases FU2 vs. Controls FU2		
	Years	Mean	Mean	Mean	Mean ∆ (95%CI)	*p*	CR *	Mean ∆ (95%CI)	*p*	CR
Fatigue	≤58	37.67	29.64	24.74	−8.03 (−10.43, −5.62)	<0.0001	s	4.90 (2.09, 7.71)	0.01	s
	59–64	31.53	30.46	29.02	−1.07 (−3.15, 1.02)	0.34		1.44 (−1.34, 4.22)	0.91	
	≥64	37.64	38.01	35.92	0.37 (−1.86, 2.60)	0.65		2.09 (−5.15, 0.98)	0.72	
Pain	≤58	29.61	26.32	25.38	−3.29 (−6.33, −0.25)	0.013	s	0.94 (−2.63, 4.51)	1	
	59–64	25.62	25.72	29.2	0.11 (−2.72, 2.94)	0.83		−3.37 (−6.90, 0.16)	0.43	
	≥64	31.11	33.33	36.08	2.23 (−0.77, 5.23)	0.16		−2.70 (−6.70, 1.31)	0.72	
Nausea/Vomiting	≤58	4.72	4.82	3.3	0.10 (−1.36, 1.55)	0.97		1.52 (0.10, 2.93)	0.24	
	59–64	3.38	3.15	2.91	−0.23 (−1.61, 1.14)	0.88		0.23 (−0.94, 1.41)	1	
	≥64	3.67	4.09	3.65	0.42 (−1.07, 1.91)	0.84		0.42 (−0.99, 1.83)	0.99	
Dyspnoe	≤58	28.2	24.18	17.76	4.07 (−7.09, −1.05)	0.005	s	6.42 (3.26, 9.59)	0.004	s
	59–64	23.7	25.65	22.43	1.94 (−1.07, 4.96)	0.29		3.25 (−0.06, 6.56)	0.37	
	≥64	26.41	26.72	26.22	0.31 (−2.59, 3.22)	0.93		0.55 (−3.16, 4.26)	1	
Insomnia	≤58	47.12	41.91	33.68	−5.21 (−8.72, −1.71)	0.0074	s	8.45 (4.40, 12.49)	0.0008	s
	59–64	44.96	41.97	34.38	2.99 (−6.39, 0.41)	0.0297	t	7.72 (3.87, 11.56)	0.001	s
	≥64	40.56	42.46	36.01	1.90 (−1.40, 5.20)	0.234		6.32 (2.16, 10.48)	0.03	s
Appetite Loss	≤58	8.89	6.38	4.77	−2.51 (−4.72, −0.31)	0.02	s	1.61 (−0.23, 3.44)	0.73	
	59–64	4.69	6.05	6.9	1.36 (−0.65, 3.37)	0.3027		−0.85 (−2.99, 1.19)	0.97	
	≥64	7.66	10.7	10.82	3.03 (0.40, 5.66)	0.04	s	−0.03 (−2.77, 2.71)	1	
Constipation	≤58	10.69	10.4	7.76	−0.29 (−2.74, 2.16)	0.81		2.62 (0.24, 5.00)	0.44	
	59–64	11.55	13.21	10.11	1.66 (−0.67, 4.00)	0.15		3.07 (0.42, 5.71)	0.2	
	≥64	15.72	15.63	14.21	−0.09 (−2.84, 2.66)	0.74		1.43 (−1.73, 4.59)	0.91	
Diarrhea	≤58	9.84	10.08	8.39	0.24 (−2.11, 2.60)	0.9		1.66 (−0.82, 4.13)	0.73	
	59–64	9.52	7.32	7.3	−2.20 (−4.31, −0.08)	0.071		0.11 (−2.04, 2.26)	1	
	≥64	8.2	7.21	6.84	−0.99 (−3.25, 1.27)	0.41		0.25 (−2.47, 1.97)	1	
Financial Difficulties	≤58	24.98	11.53	5.5	−13.45 (−16.52 −10.38)	<0.0001	l	6.02 (3.95, − 8.45)	<0.0001	s
	59–64	8.29	7.23	6.06	−1.06 (−2.83, 0.71)	0.1862		1.17 (−0.89, 3.23)	0.91	
	≥64	12.74	8.82	7.94	−3.92 (−6.29, −1.55)	0.0053	s	1.10 (−1.41, 3.61)	0.94	

* Clinical relevance (CR): tirival (t), small (s), medium (m), large (l).

## Data Availability

Data cannot be made publicly available for legal reasons. Due to data privacy rules and according to German law (§ 75 SGB X) access to the data is granted only to responsible scientific personnel at DKFZ, Heidelberg, Germany, and UKE, Hamburg, Germany within the framework of the respective research project. It is not permitted to give third parties access to the data without a research proposal approved by the principal investigator.
